# Multicenter cohort study reveals: composite inflammatory indexs are associated with increased risk of diabetes in patients with hypertension

**DOI:** 10.3389/fendo.2026.1819071

**Published:** 2026-04-21

**Authors:** Fengyou Yao, Junqi Gou, Xiao Zhu, Kai Tang, Chaohui Liu

**Affiliations:** 1 Geriatric Diseases Institute of Chengdu/Cancer Prevention and Treatment Institute of Chengdu, Department of Cardiology, Chengdu Fifth People’s Hospital (The Second Clinical Medical College, Affiliated Fifth People’s Hospital of Chengdu University of Traditional Chinese Medicine), Chengdu, China; 2 Department of Cardiology, Chengdu Integrated Traditional Chinese Medicine &Western Medicine Hospital, Chengdu First People’s Hospital, Chengdu, China; 3 Department of Cardiology, Suining Central Hospital, Suining, China

**Keywords:** composite inflammatory index, diabetes, hypertension, insulin resistance, metabolic disorder

## Abstract

**Background:**

Patients with hypertension commonly exhibit a persistent chronic inflammatory state. Accumulating evidence suggests that inflammation can induce insulin resistance, which is a key pathological mechanism in the development of diabetes. However, whether composite inflammatory indices are independently associated with the risk of incident diabetes in hypertensive patients remains insufficiently supported by current evidence.

**Methods:**

Multivariable-adjusted Cox proportional hazards regression models were used to assess the associations between composite inflammatory indices and the risk of incident diabetes in patients with hypertension. Kaplan-Meier (KM) curves were generated to visually depict the cumulative incidence of diabetes across different levels of these inflammatory indices. Furthermore, comparative analyses were conducted to identify the most predictive composite inflammatory marker.

**Results:**

Cox regression analysis revealed that elevated composite inflammatory indices were significantly associated with an increased risk of incident diabetes in patients with hypertension. KM curves further demonstrated that individuals with higher inflammatory levels exhibited a significantly higher cumulative incidence of diabetes during follow-up compared to those with lower levels. Moreover, comparative analyses among the inflammatory markers identified the inflammatory burden index (IBI) as the most effective predictor of diabetes risk.

**Conclusion:**

This study demonstrates that elevated composite inflammatory indices are closely associated with an increased risk of future diabetes in patients with hypertension, with a notable threshold effect. These findings suggest that actively controlling inflammatory levels may help reduce the risk of diabetes in this population. Furthermore, the IBI holds promise as a simple and accessible risk assessment tool for screening and early identification of individuals at high risk for diabetes.

## 1 Introduction

Diabetes is a particularly common chronic and prevalent disease, with a notably high incidence rate (1, 2). Long-term poor blood glucose control can lead to damage to the cardiovascular system, kidneys, peripheral blood vessels, and nervous system, and may even progress to kidney failure or cardiovascular death (3–5). Moreover, with rising obesity rates, changes in lifestyle habits, and increased life expectancy, the prevalence of diabetes is gradually increasing (6, 7). This has become a significant threat to public health, and the associated societal burden is growing particularly fast. Therefore, sufficient attention should be given to early risk assessment, timely intervention, and prevention of diabetes.

Hypertension, a major cardiovascular disease and a particularly common chronic condition, shares similarities with diabetes in its prevalence and long-term impact (8–10). Inadequate blood pressure control over time not only impairs cardiac function but also promotes plaque formation in blood vessels, leading to cardiovascular diseases such as coronary heart disease, as well as hypertension-related kidney damage (11–13). Notably, when hypertension coexists with diabetes, the burden of target organ damage is significantly exacerbated, with cardiovascular complications being the most severe (14, 15). The combination of these two conditions tends to create a worsening cycle, resulting in more serious adverse events (15, 16). Therefore, preventing the onset of diabetes in hypertensive patients is of critical importance.

In the prevention of diabetes among hypertensive patients, previous efforts have predominantly focused on dietary management—particularly low-sugar diets—avoiding obesity, and controlling lipid levels as much as possible (17–19). However, some patients still develop diabetes even when these traditional risk factors are well-managed, highlighting the need to explore whether other risk factors also play a significant role in the onset of diabetes in this population. Recently, emerging research has increasingly emphasized the important role of inflammation in this process (20–22). Studies indicate that long-term chronic inflammation may impair pancreatic β-cell function, while the release of inflammatory cytokines can induce insulin resistance and reduce glucose tolerance (21–24). The combined effect of these mechanisms plays a crucial role in the development of diabetes. Moreover, hypertensive patients often experience a sustained state of low-grade chronic inflammation, which may further create a conducive environment for the onset of diabetes (25–27).

Given the role of inflammation in various diseases, traditional single inflammatory markers may not sufficiently reflect the body’s true inflammatory status. Consequently, several composite inflammatory indices have been developed, such as the platelet-to-lymphocyte ratio (PLR), pan-immune-inflammation value (PIV), and inflammatory burden index (IBI), which are now widely used (28–31). These indices integrate multiple blood cell counts from routine blood tests or combine them with C-reactive protein (CRP) to provide a more comprehensive reflection of the body’s inflammatory state (29, 31). Moreover, these composite inflammatory indices have demonstrated excellent performance in predicting disease risk and adverse outcomes across various conditions (28, 32–34). Studies have indicated that PIV, compared to single traditional inflammatory markers, may be more closely associated with arterial calcification and can predict early mortality in patients with sepsis, highlighting its potential clinical significance (32, 34). Similarly, in cancer patients, research suggests that the inflammatory burden represented by IBI may serve as a prognostic biomarker (33). In summary, the emergence of these composite inflammatory markers offers a more comprehensive, simple, and practical tool for assessing systemic inflammation.

Given the critical role of inflammation in the development of diabetes and the excellent performance demonstrated by composite inflammatory indices, this study aims to comprehensively evaluate the relationship between composite inflammatory indices and diabetes in hypertensive patients through a multicenter cohort design. The findings will provide evidence supporting the important role of these composite inflammatory indicators in diabetes risk assessment among individuals with hypertension.

## 2 Material and methods

### 2.1 Study population

**Figure 1:**
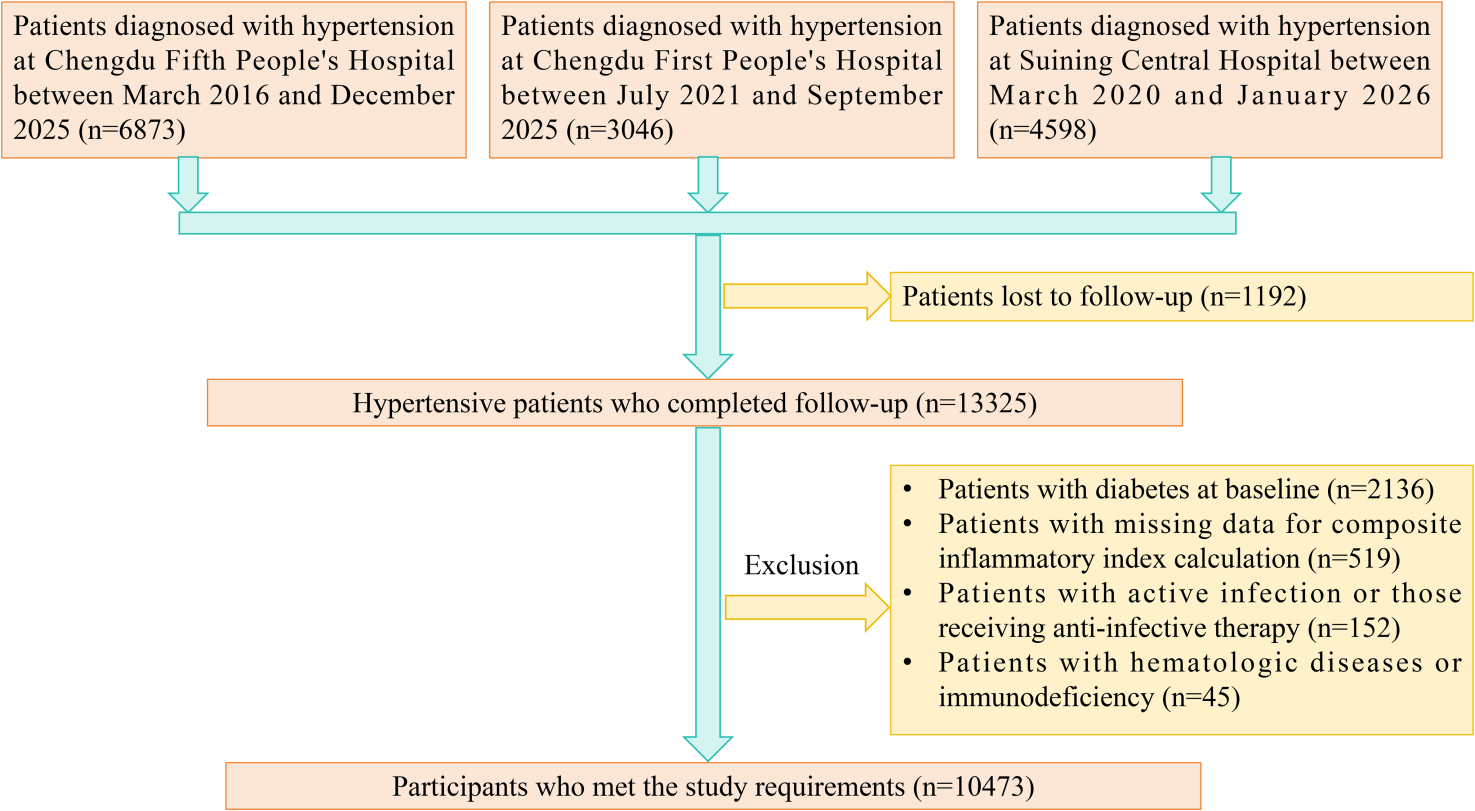
Study population selection.

This multicenter cohort study initially enrolled 14517 patients with hypertension from three centers: Chengdu Fifth People’s Hospital, Chengdu First People’s Hospital, and Suining Central Hospital. Among these, 13325 patients completed follow-up. After further excluding patients with prevalent diabetes at baseline, missing data for composite inflammatory index calculation, active infection or use of anti-infective agents, as well as those with hematologic diseases or immunodeficiency, a total of 10,473 patients were included in the final analysis (**Figure 1**).

This study was conducted in strict accordance with clinical research regulations and was approved by the ethics committees of the three participating centers: Chengdu Fifth People’s Hospital (Approval No. NX20160304), Chengdu First People’s Hospital (Approval No. CY20210712), and Suining Central Hospital (Approval No. X20200317). Written informed consent was obtained from all participants prior to enrollment, after they were fully informed of the study details.

### 2.2 Covariates and data collection

The data collection for this study encompasses demographic information, medical history, medication use, and laboratory measurements. Data are obtained through multiple modalities to ensure comprehensive coverage, including electronic medical records from healthcare centers, medical insurance records, and periodic in-person interviews or telephone follow-ups. Anthropometric and vital sign measurements, such as blood pressure, height, and weight, are collected by qualified personnel or through standardized measurement procedures. Blood samples were collected after an overnight fast to ensure standardization and to minimize the influence of dietary intake on biochemical parameters. Venous blood samples were obtained by trained nurses using standardized phlebotomy techniques (35–37).

Biochemical indicators are derived from routine laboratory test results, covering complete blood count, liver function tests, lipids, cholesterol, fasting plasma glucose (FPG) and CRP, among others. Information on disease history and medication use includes conditions such as coronary heart disease and hyperlipidemia, as well as the use of various medications, including lipid-lowering agents, antiplatelet drugs, diuretics, beta-blockers, calcium channel blockers, and ACE inhibitors or ARBs.

### 2.3 Acquisition of the composite inflammation indexs

The composite inflammatory indices examined in this study include the PLR, the PIV, and the IBI. Their specific calculation formulas are as follows (28, 29, 31):


PLR = Platelet count/Lymphocyte count;

$$PIV\;=\;\left(Neutrophil\;count\;\times \;Platelet\;count\;\times \;Monocyte\;count\right)/Lymphocyte\;count;$$

IBI = C−reactive protein × Neutrophil count/Lymphocyte count.

### 2.4 Study endpoint

The endpoint of this study is the incident diagnosis of diabetes in hypertensive patients during follow-up. Diabetes is diagnosed in strict accordance with current endocrinology society guidelines, based on a comprehensive assessment that includes fasting blood glucose, oral glucose tolerance test (OGTT), glycated hemoglobin (HbA1c) levels, and relevant clinical symptoms (38–40). Follow-up time is defined as the period until the earliest of the following occurrences: the date of diabetes diagnosis, the last valid follow-up visit, or the predetermined study cutoff date.

### 2.5 Statistical analysis

Patients were divided into two groups based on the occurrence of diabetes, and their baseline characteristics were compared. According to data distribution, continuous variables were expressed as mean ± standard deviation (SD) or median (interquartile range), while categorical variables were presented as numbers (percentages). Subsequently, a series of four gradually adjusted Cox regression models were used to analyze the relationship between composite inflammatory markers and diabetes risk in hypertensive patients, with separate analyses for continuous and categorical variables. To further explore the dose-response relationship, restricted cubic splines (RCS) were employed to evaluate the association patterns between composite inflammatory indices and diabetes risk, with threshold effect analysis based on inflection points.

Furthermore, the predictive performance of different inflammatory indices for diabetes was compared using the area under the receiver operating characteristic (ROC) curve (AUC) and decision curve analysis (DCA). In addition, the integrated discrimination improvement (IDI), net reclassification improvement (NRI), and C-index were applied to assess the incremental predictive value of these inflammatory indicators.

All statistical analyses were conducted using R software (version 4.2.3), with statistical significance defined as a two-sided P-value < 0.05.

## 3 Results

### 3.1 Comparison of two groups of baseline characteristics

Among the 10473 hypertensive patients included in this study, a total of 2219 developed diabetes during the longest 8.5-year follow-up period. **Table 1** shows the comparison of baseline characteristics between the two groups. Compared to the non-diabetic group, patients in the diabetic group were older, had higher body mass index (BMI), and were more likely to be current smokers and alcohol consumers. Their blood pressure levels were also comparatively higher.

**Table 1:** Baseline characteristics of the study population.

Characteristic	Overall	Non-Diabetes	Diabetes	P value
Number	10473	8254	2219	
Sex (%)				0.087
Female	5483 (52.35%)	4357 (52.79%)	1126 (50.74%)	
Male	4990 (47.65%)	3897 (47.21%)	1093 (49.26%)	
Age (years)	58.67±8.31	58.52±8.29	59.22±8.33	<0.001
BMI (kg/m^2^)	25.87±3.99	25.71±3.99	26.45±3.94	<0.001
Current smoking (%)	2627 (25.08%)	2018 (24.45%)	609 (27.44%)	0.004
Current drinking (%)	2432 (23.22%)	1879 (22.76%)	553 (24.92%)	0.033
SBP (mmHg)	146.75±18.94	145.47±18.33	151.51±20.33	<0.001
DBP (mmHg)	88.66±13.80	87.84±13.53	91.68±14.39	<0.001
Laboratory tests				
TSH (uIU/mL)	2.20 (1.47-3.28)	2.18 (1.47-3.30)	2.23 (1.49-3.12)	0.744
ALT (U/L)	17.30 (12.00-28.00)	17.16 (12.35-28.00)	17.60 (12.00-27.03)	0.486
AST (U/L)	18.32 (15.00-24.00)	18.09 (15.00-24.00)	18.84 (15.00-24.00)	0.558
TC (mmol/L)	4.14±0.94	4.10±0.91	4.30±1.05	<0.001
TG (mmol/L)	0.68 (0.56-1.55)	0.66 (0.55-1.55)	0.73 (0.62-1.55)	0.017
HDL-C (mg/dL)	2.77±0.81	2.75±0.80	2.84±0.83	<0.001
LDL-C (mg/dL)	1.16±0.28	1.17±0.29	1.14±0.27	<0.001
FPG (mmol/L)	4.76±0.91	4.74±0.92	4.81±0.87	0.001
CRP (mg/L)	6.43 (4.67-9.60)	5.96 (4.59-8.95)	8.39 (5.44-11.56)	<0.001
Composite inflammatory indexs				
PLR	130.36±46.03	128.03±48.86	139.02±32.02	<0.001
PIV	123.79±46.29	119.21±48.70	140.81±30.38	<0.001
IBI	8.12±7.23	7.96±7.72	8.69±4.93	<0.001
Medical history and medications				
CHD (%)	3288 (31.40%)	2540 (30.77%)	748 (33.71%)	0.008
Hyperlipidemia (%)	4847 (46.28%)	3770 (45.67%)	1077 (48.54%)	0.016
Lipid-lowering drugs (%)	1870 (17.86%)	1415 (17.14%)	455 (20.50%)	<0.001
Antiplatelet drugs (%)	1218 (11.63%)	920 (11.15%)	298 (13.43%)	0.003
Diuretics (%)	1602 (15.30%)	1272 (15.41%)	330 (14.87%)	0.531
Beta-blockers (%)	2311 (22.07%)	1812 (21.95%)	499 (22.49%)	0.59
ACEIs/ARBs (%)	4396 (41.97%)	3406 (41.26%)	990 (44.61%)	0.005
Calcium channel blockers (%)	5356 (51.14%)	4170 (50.52%)	1186 (53.45%)	0.014

Data are presented as mean ± standard deviation, median (interquartile range), or as numbers, and percentages.

BMI, body mass index; SBP, systolic blood pressure; DBP, diastolic blood pressure; TSH, thyroid-stimulating hormone; ALT, alanine transaminase; AST, aspartate transaminase; HDL-C, high-density lipoprotein cholesterol; LDL-C, low-density lipoprotein cholesterol; TC, total cholesterol; TG, triglyceride; FPG, fasting plasma glucose; CRP, c-reactive protein; PLR, platelet-to-lymphocyte ratio; PIV, pan-immune-inflammation valu; IBI, inflammatory burden index; CHD, coronary heart disease; ACEIs, angiotensin-converting enzyme inhibitors; ARBs, angiotensin receptor blockers.

**Figure 2:**
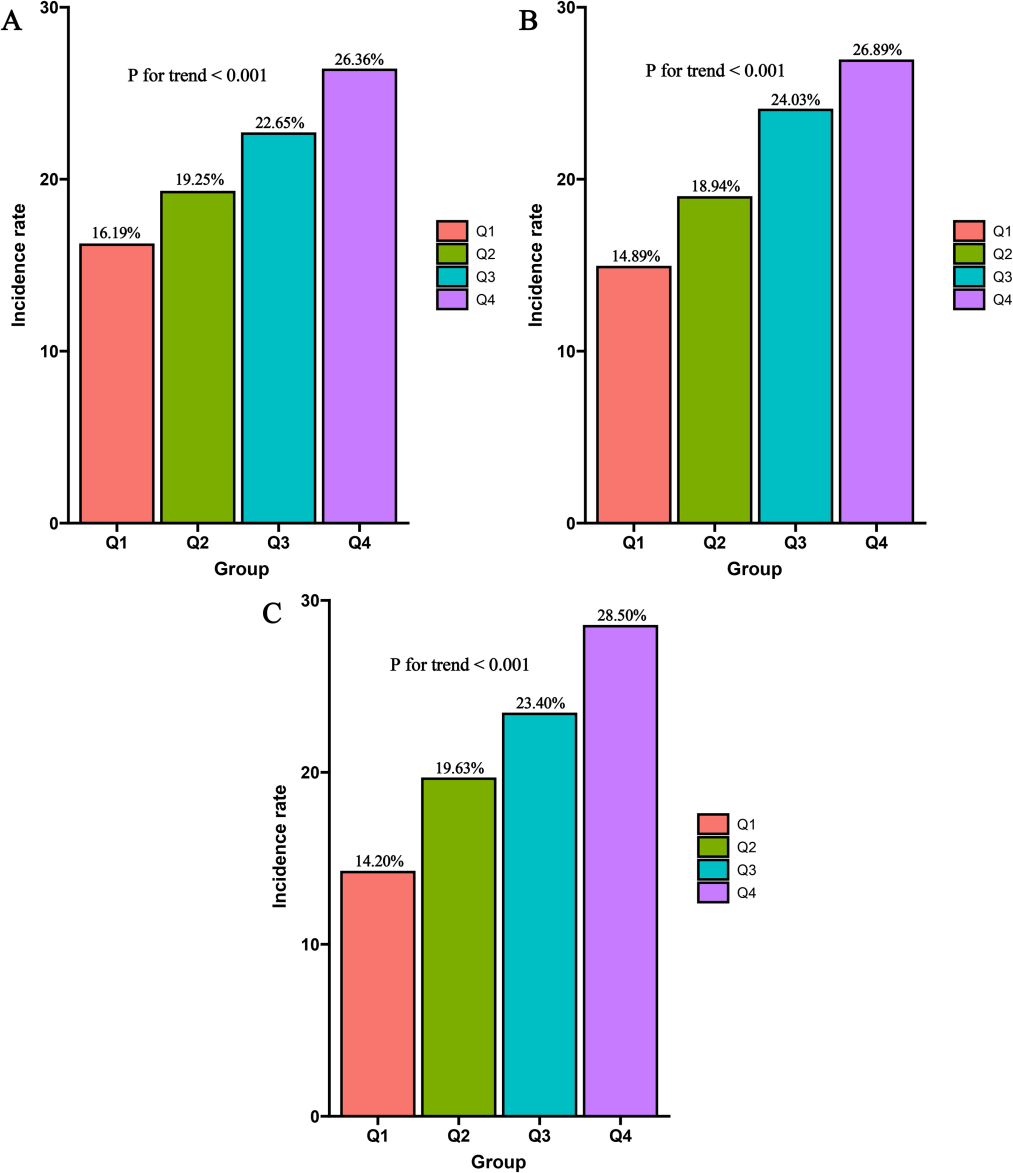
Comparison of diabetes prevalence between groups. **(A)** PLR; **(B)** PIV; **(C)** IBI.

In terms of biochemical profiles, the diabetic group exhibited significantly elevated levels of total cholesterol, low-density lipoprotein cholesterol, and FPG, while high-density lipoprotein cholesterol was lower. CRP levels were also notably higher in this group. Furthermore, all three composite inflammatory indexs (PLR, PIV, and IBI) were significantly elevated in the diabetic group compared to the non-diabetic group. Regarding comorbidities and medication use, the diabetic group had a higher prevalence of coronary heart disease and hyperlipidemia. They were also more frequently prescribed lipid-lowering drugs, antiplatelet drugs, angiotensin-converting enzyme inhibitors/angiotensin II receptor blockers (ACEIs/ARBs), and calcium channel blockers. More importantly, when patients were stratified into four groups based on quartiles of the three composite inflammatory indices, the incidence of diabetes showed a gradual increase from Q1 to Q4 across all indices (**Figure 2**).

### 3.2 Elevated composite inflammatory indexs are associated with an increased risk of diabetes in hypertensive patients

A multi-model stepwise adjusted Cox regression analysis was performed to assess the association between three composite inflammatory indices and diabetes risk in hypertensive patients. As summarized in **Table 2**, all indices consistently showed a significant positive association with incident diabetes.

**Table 2:** Relationship of composite inflammatory indices with diabetes risk in hypertensive patients.

Diabetes	Model 1	Model 2	Model 3	Model 4
	HR (95% CI) P	HR (95% CI) P	HR (95% CI) P	HR (95% CI) P
PLR				
PLR (per 1SD increase)	2.917 [2.634, 3.230] <0.001	2.904 [2.632, 3.203] <0.001	2.891 [2.612, 3.199] <0.001	2.867 [2.596, 3.166] <0.001
Quartiles of PLR				
Q1	Reference	Reference	Reference	Reference
Q2	1.351 [1.187, 1.538] <0.001	1.349 [1.186, 1.534] <0.001	1.341 [1.178, 1.525] <0.001	1.338 [1.176, 1.523] <0.001
Q 3	1.727 [1.525, 1.955] <0.001	1.716 [1.512, 1.947] <0.001	1.701 [1.501, 1.928] <0.001	1.695 [1.495, 1.921] <0.001
Q 4	3.158 [2.797, 3.567] <0.001	3.130 [2.764, 3.545] <0.001	3.108 [2.748, 3.515] <0.001	3.095 [2.735, 3.501] <0.001
P for trend	<0.001	<0.001	<0.001	<0.001
PIV				
PIV (per 1SD increase)	2.115 [1.995, 2.242] <0.001	2.090 [1.973, 2.215] <0.001	2.088 [1.973, 2.210] <0.001	2.088 [1.971, 2.211] <0.001
Quartiles of PIV				
Q1	Reference	Reference	Reference	Reference
Q2	1.490 [1.303, 1.703] <0.001	1.481 [1.297, 1.691] <0.001	1.475 [1.291, 1.685] <0.001	1.472 [1.288, 1.682]] <0.001
Q 3	2.054 [1.810, 2.332] <0.001	2.050 [1.802, 2.332] <0.001	2.027 [1.784, 2.303] <0.001	2.018 [1.776, 2.293] <0.001
Q 4	3.594 [3.171, 4.072] <0.001	3.581 [3.152, 4.068] <0.001	3.546 [3.126, 4.024] <0.001	3.533 [3.113, 4.010] <0.001
P for trend	<0.001	<0.001	<0.001	<0.001
IBI				
IBI (per 1SD increase)	1.312 [1.261, 1.365] <0.001	1.302 [1.251, 1.356] <0.001	1.302 [1.249, 1.356] <0.001	1.301 [1.249, 1.355] <0.001
Quartiles of IBI				
Q1	Reference	Reference	Reference	Reference
Q2	1.624 [1.420, 1.857] <0.001	1.613 [1.411, 1.844] <0.001	1.607 [1.405, 1.837] <0.001	1.604 [1.403, 1.834] <0.001
Q 3	2.096 [1.838, 2.390] <0.001	2.094 [1.840, 2.383] <0.001	2.070 [1.818, 2.357] <0.001	2.061 [1.810, 2.348] <0.001
Q 4	3.885 [3.424, 4.409] <0.001	3.881 [3.411, 4.415] <0.001	3.839 [3.378, 4.363] <0.001	3.824 [3.364, 4.347] <0.001
P for trend	<0.001	<0.001	<0.001	<0.001

Model 1, no covariates were adjusted.

Model 2, age, sex, BMI, smoking status, drinking status, SBP, and DBP were adjusted.

Model 3, Model 2 plus adjustment for TSH, ALT, AST, TC, TG, HDL.C, LDL.C, and FPG.

Model 4, Model 3 plus adjustment for CHD, Hyperlipidemia, Lipid-lowering drugs, Antiplatelet drug, diuretics, beta-blockers, ACEIs/ARBs, and calcium channel blockers.

PLR, platelet-to-lymphocyte ratio; PIV, pan-immune-inflammation valu; IBI, inflammatory burden index; HR, hazard ratio; CI, confidence interval.

Other abbreviations, see **Table 1**.

In the unadjusted model (Model 1), each 1-SD increase in PLR, PIV, and IBI was associated with a 191.7% (hazard ratio [HR]: 2.917, 95% confidence interval [CI]: 2.634–3.230), 111.5% (HR: 2.115, 95% CI: 1.995–2.242), and 31.2% (HR: 1.312, 95% CI: 1.261–1.365) increase in diabetes risk, respectively (**Table 2**). These associations remained robust in the fully adjusted model (Model 4), with corresponding risk increases of 186.7% for PLR, 108.8% for PIV, and 30.1% for IBI per 1-SD increment (**Table 2**).

When analyzed by quartiles, a clear dose-response relationship was observed. Compared with the lowest quartile (Q1), diabetes risk progressively increased across Q2 to Q4, with the highest quartile (Q4) demonstrating substantially elevated HRs of 3.095 (95% CI: 2.735–3.501) for PLR, 3.533 (95% CI: 3.113–4.010) for PIV, and 3.824 (95% CI: 3.364–4.347) for IBI (**Table 2**).

**Figure 3:**
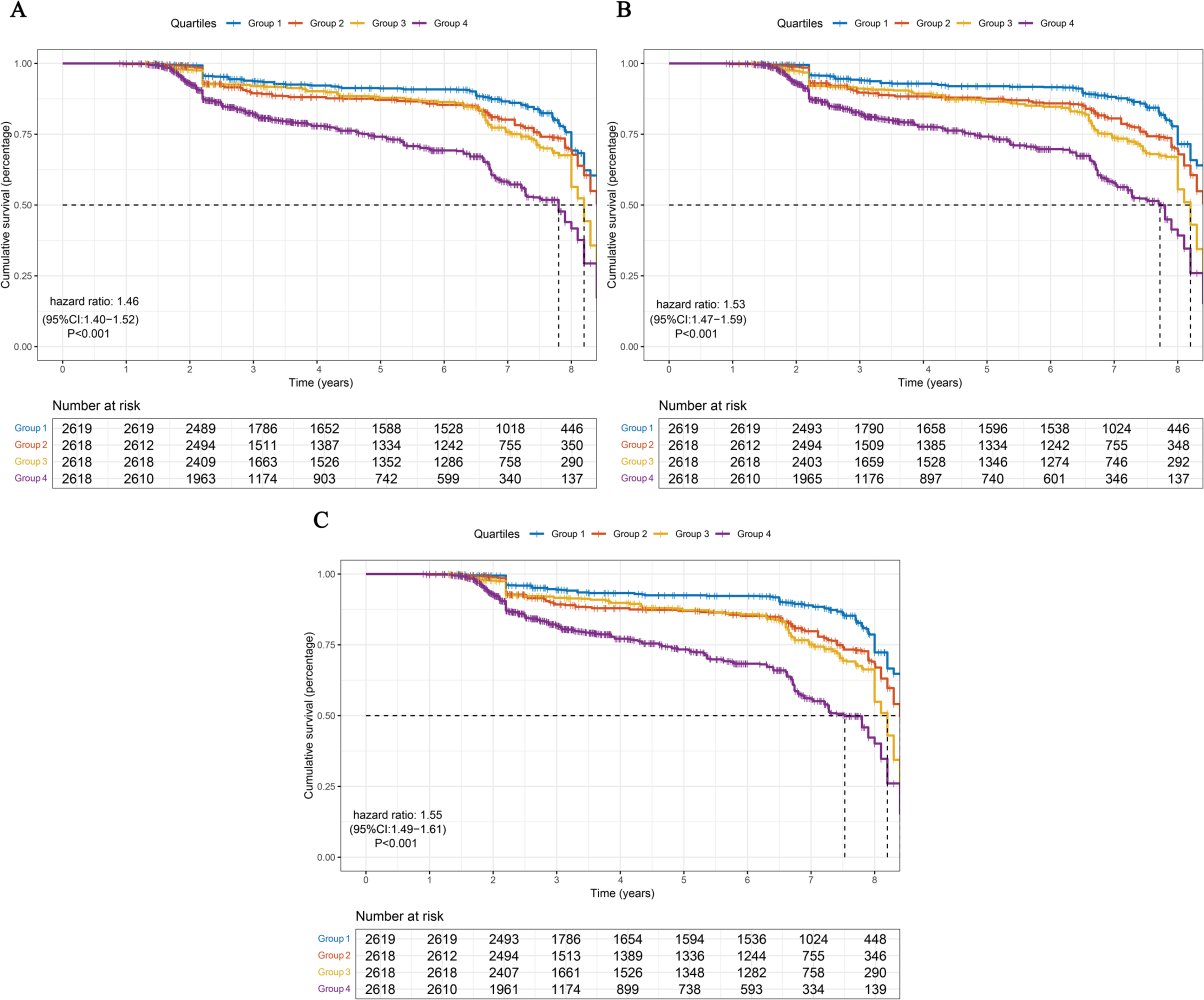
Kaplan-Meier curves for cumulative incidence of diabetes during follow-up between groups **(A)** PLR; **(B)** PIV; **(C)** IBI Group1: Q1; Group2: Q2; Group3: Q3; Group4: Q4.

Consistent with these findings, Kaplan–Meier curves (**Figure 3**) visually confirmed the graded increase in diabetes risk from Q1 to Q4, with statistically significant differences observed across all quartile groups.

### 3.3 Threshold effects of composite inflammatory indices on diabetes

**Figure 4:**
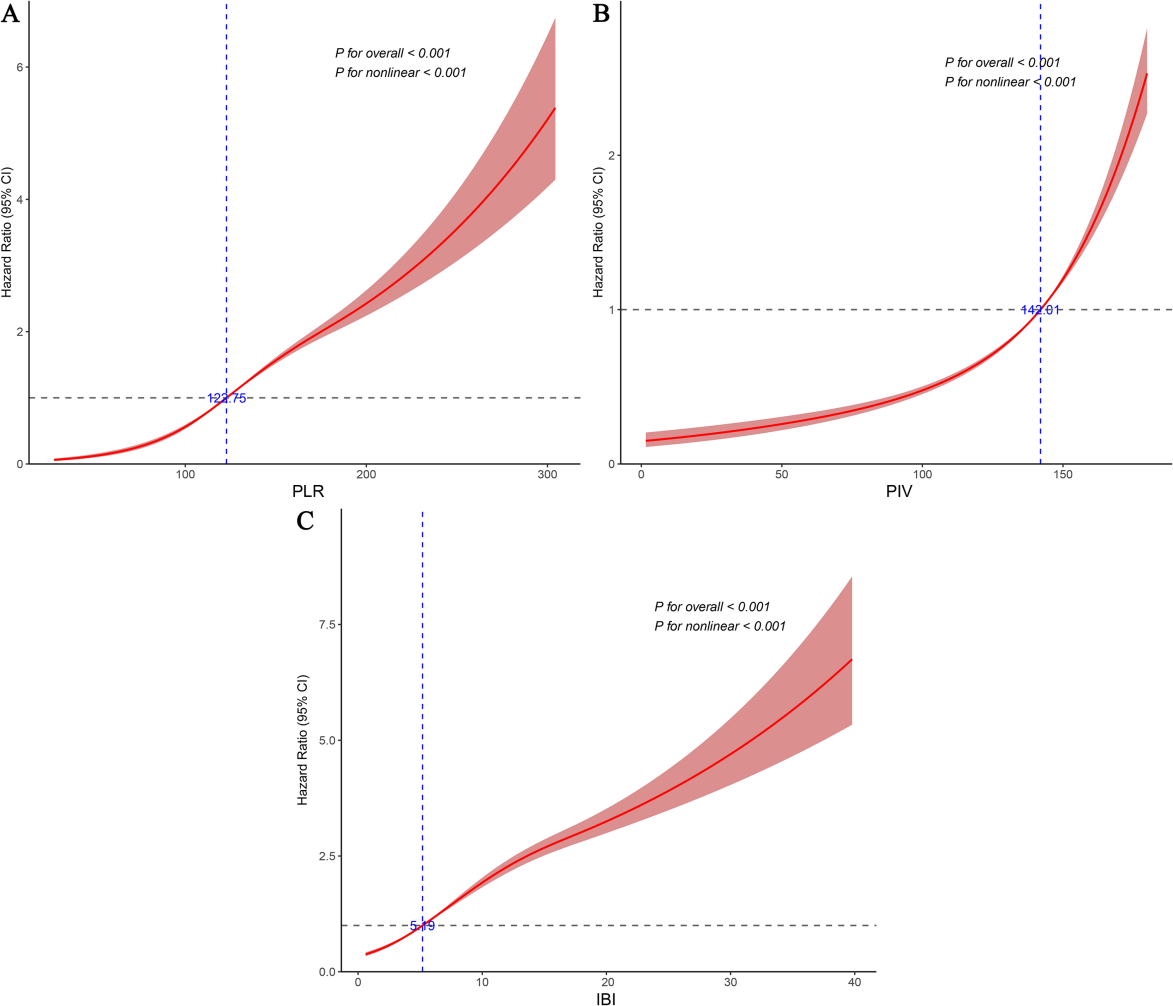
Dose-response relationships between different composite inflammatory indices and diabetes risk. **(A)** PLR; **(B)** PIV; **(C)** IBI.

Given that Cox regression analysis indicated a significant dose-response relationship between composite inflammatory indices and diabetes risk, we further employed RCS to evaluate their potential nonlinear association and threshold effects. The results revealed that all three composite inflammatory indices (PLR, PIV, and IBI) exhibited significant nonlinear relationships with diabetes risk in hypertensive patients, with identified threshold values of 122.75 for PLR, 142.01 for PIV, and 5.19 for IBI (**Figure 4**). Beyond these thresholds, the risk of diabetes increased more sharply (**Figure 4**).

To further clarify the high-risk profile of patients exceeding these thresholds, we conducted a two-stage comparative analysis based on the identified cutoffs. The results showed that patients with index levels above the thresholds had significantly higher diabetes risks compared to those below, with HRs of 2.189 (95% CI: 2.006–2.389) for PLR, 2.193 (95% CI: 2.010–2.393) for PIV, and 2.166 (95% CI: 1.985–2.363) for IBI (**Table 3**). Furthermore, we used data from Suining Central Hospital as an external dataset to validate the threshold, and the results were largely consistent with those from the overall analysis (**Supplementary Table 1**). In addition, we considered potential differences across age and BMI subgroups. Accordingly, we stratified the data by age and BMI and performed the same threshold validation, and the results remained robust (**Supplementary Tables S2, S3**).

**Table 3:** Threshold analysis of composite inflammation indexs and diabetes risk in hypertensive patients.

Diabetes	Model 1	Model 2	Model 3	Model 4
	HR (95% CI) P	HR (95% CI) P	HR (95% CI) P	HR (95% CI) P
PLR				
Turning point (ng/dL)	122.75	122.75	122.75	122.75
<= 122.75	Reference	Reference	Reference	Reference
> 122.75	2.229 [2.045, 2.429] <0.001	2.209 [2.023, 2.412] <0.001	2.201 [2.018, 2.401] <0.001	2.189 [2.006, 2.389] <0.001
PIV				
Turning point (ng/dL)	142.01	142.01	142.01	142.01
<= 142.01	Reference	Reference	Reference	Reference
> 142.01	2.232 [2.048, 2.433] <0.001	2.213 [2.026, 2.417] <0.001	2.205 [2.021, 2.405] <0.001	2.193 [2.010, 2.393] <0.001
IBI				
Turning point (ng/dL)	5.19	5.19	5.19	5.19
<= 5.19	Reference	Reference	Reference	Reference
> 5.19	2.206 [2.024, 2.404] <0.001	2.185 [2.001, 2.387] <0.001	2.178 [1.997, 2.376] <0.001	2.166 [1.985, 2.363] <0.001

Model 1, no covariates were adjusted. .

Model 2, age, sex, BMI, smoking status, drinking status, SBP, and DBP were adjusted.

Model 3, Model 2 plus adjustment for TSH, ALT, AST, TC, TG, HDL.C, LDL.C, and FPG.

Model 4, Model 3 plus adjustment for CHD, Hyperlipidemia, Lipid-lowering drugs, Antiplatelet drug, diuretics, beta-blockers, ACEIs/ARBs, and calcium channel blockers.

PLR, platelet-to-lymphocyte ratio; PIV, pan-immune-inflammation valu; IBI, inflammatory burden index; HR, hazard ratio; CI, confidence interval.

Other abbreviations, see **Table 1**.

These findings underscore the clinical importance of maintaining inflammatory activity below specific thresholds to mitigate diabetes risk in hypertensive patients.

### 3.4 Comparison of predictive performance among the three composite inflammatory indices

**Figure 5:**
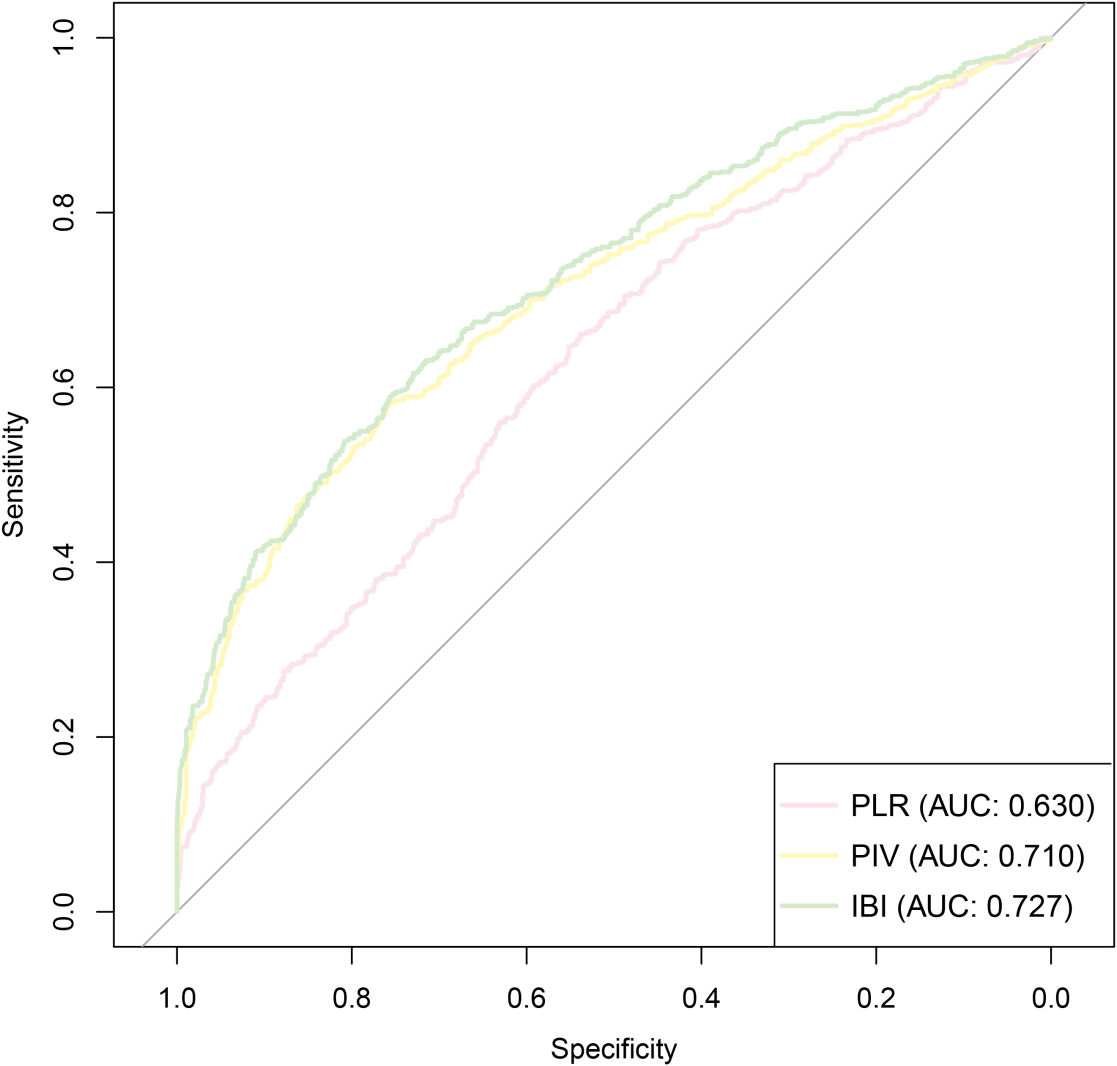
Receiver operating characteristic curves analysis of different composite inflammatory indices for diabetes risk prediction **(A)** PLR; **(B)** PIV; **(C)** IBI.

**Figure 6:**
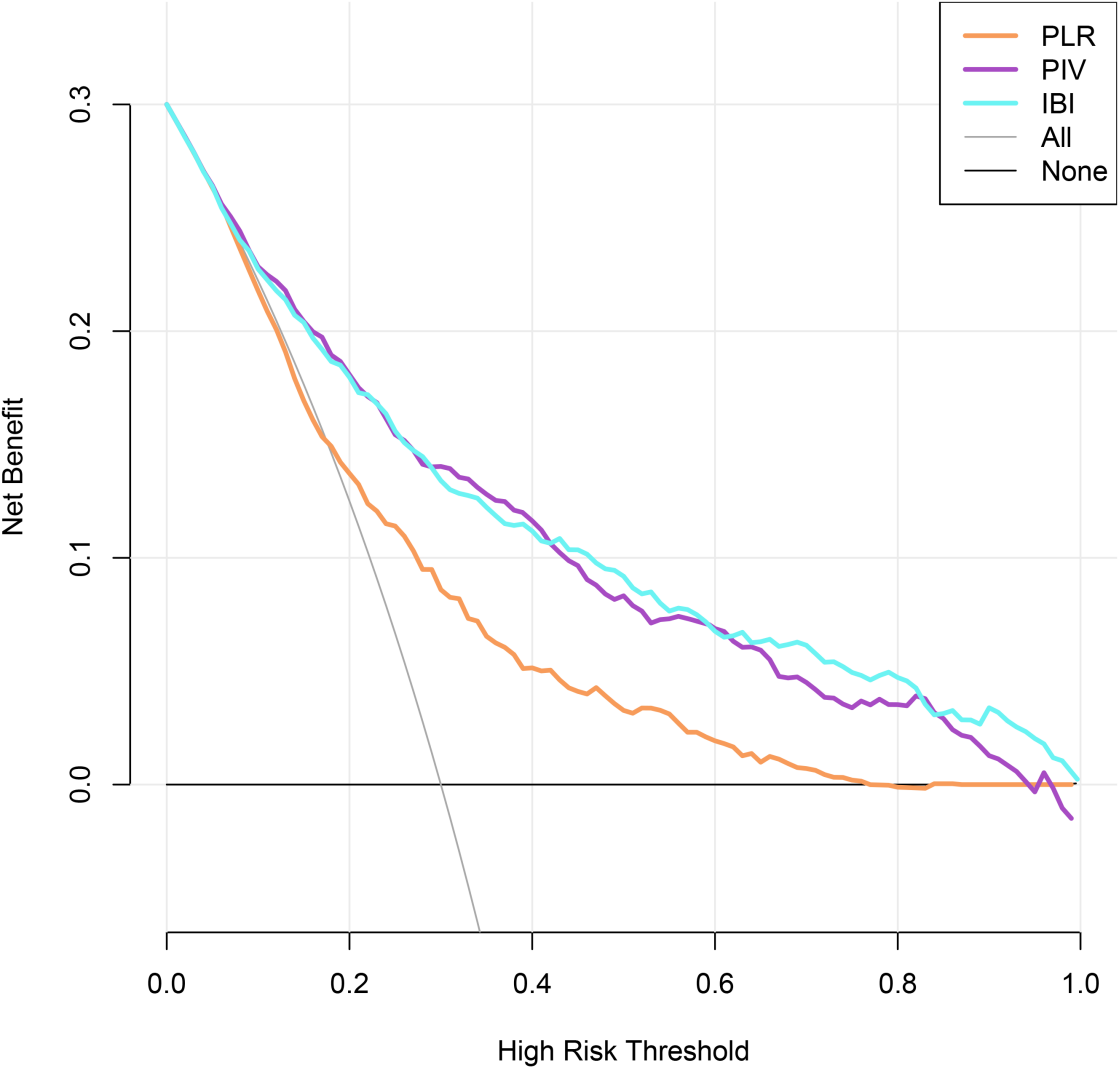
Decision curve analysis of different composite inflammatory indices for diabetes risk prediction. **(A)** PLR; **(B)** PIV; **(C)** IBI.

Given that all three composite inflammatory indices were significantly associated with diabetes risk, we further evaluated and compared their performance as predictive markers. First, ROC curve analysis showed that all three indices had good predictive ability, with AUC of 0.630 for PLR, 0.710 for PIV, and 0.727 for IBI (**Figure 5**, **Table 4**). Among them, IBI exhibited the highest AUC, indicating the strongest predictive performance (**Figure 5**, **Table 4**). DCA further supported these findings, with IBI consistently demonstrating the highest net clinical benefit (**Figure 6**).

**Table 4:** Comparative analysis of receiver operating characteristic for various composite inflammation indexs.

Composite inflammation indexs	AUC	95%CI low	95%CI up	Specificity	Sensitivity	Positive-pv	Negative-pv
PLR	0.630	0.608	0.651	0.538	0.661	0.240	0.878
PIV	0.710	0.688	0.731	0.756	0.582	0.345	0.891
IBI	0.727	0.707	0.747	0.808	0.538	0.404	0.879

PLR, platelet-to-lymphocyte ratio; PIV, pan-immune-inflammation valu; IBI, inflammatory burden index; AUC, area under the curve; Positive-pv, positive predictive value; Negative-pv, negative predictive value.

Other abbreviations, see **Table 1**.

To assess the incremental predictive value of each inflammatory index, we added PLR, PIV, and IBI separately to the fully adjusted Model 4 (**Table 5**). All indices significantly improved model performance: The IDI increased by 0.159, 0.197, and 0.261, respectively; The NRI increased by 0.345, 0.466, and 0.528, respectively; The C−index improved from a baseline of 0.712 to 0.745 (PLR), 0.778 (PIV), and 0.789 (IBI) (**Table 5**). These results consistently indicate that IBI provides the greatest improvement in predictive performance and carries the strongest independent predictive value. Therefore, IBI may serve as a practical indicator for diabetes risk stratification and early intervention in hypertensive patient.

**Table 5:** Comparison of incremental predictive value of various composite inflammation indexs for diabetes risk.

Model	IDI	p-value	Continuous NRI	p-value	C-index
	Estimate (95%CI)		Estimate (95%CI)		
Diabetes					
Model 5	Reference		Reference		0.712
Model 5+PLR	0.159 (0.095, 0.211)	<0.001	0.345 (0.227, 0.421)	<0.001	0.745
Model 5+PIV	0.197 (0.159, 0.255)	<0.001	0.466 (0.352, 0.599)	<0.001	0.778
Model 5+IBI	0.261 (0.195, 0.311)	<0.001	0.528 (0.383, 0.619)	<0.001	0.789

PLR, platelet-to-lymphocyte ratio; PIV, pan-immune-inflammation valu; IBI, inflammatory burden index; IDI, integrated discrimination improvement; NRI, net reclassification improvement.

Other abbreviations, see **Table 1**.

### 3.5 Subgroup analysis

**Figure 7:**
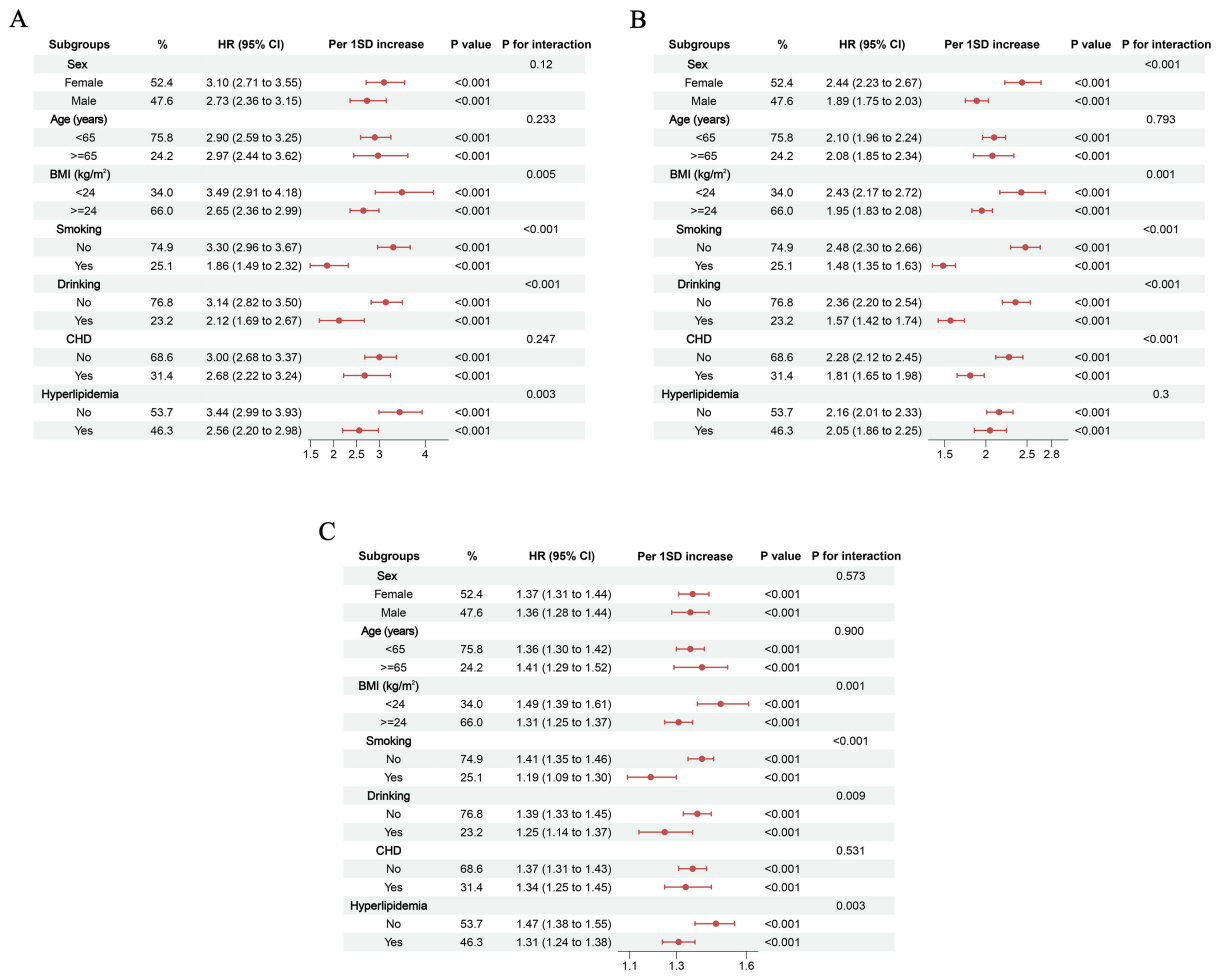
Subgroup analyses based on baseline characteristics and disease status. **(A)** PLR; **(B)** PIV; **(C)** IBI.

**Figure 8:**
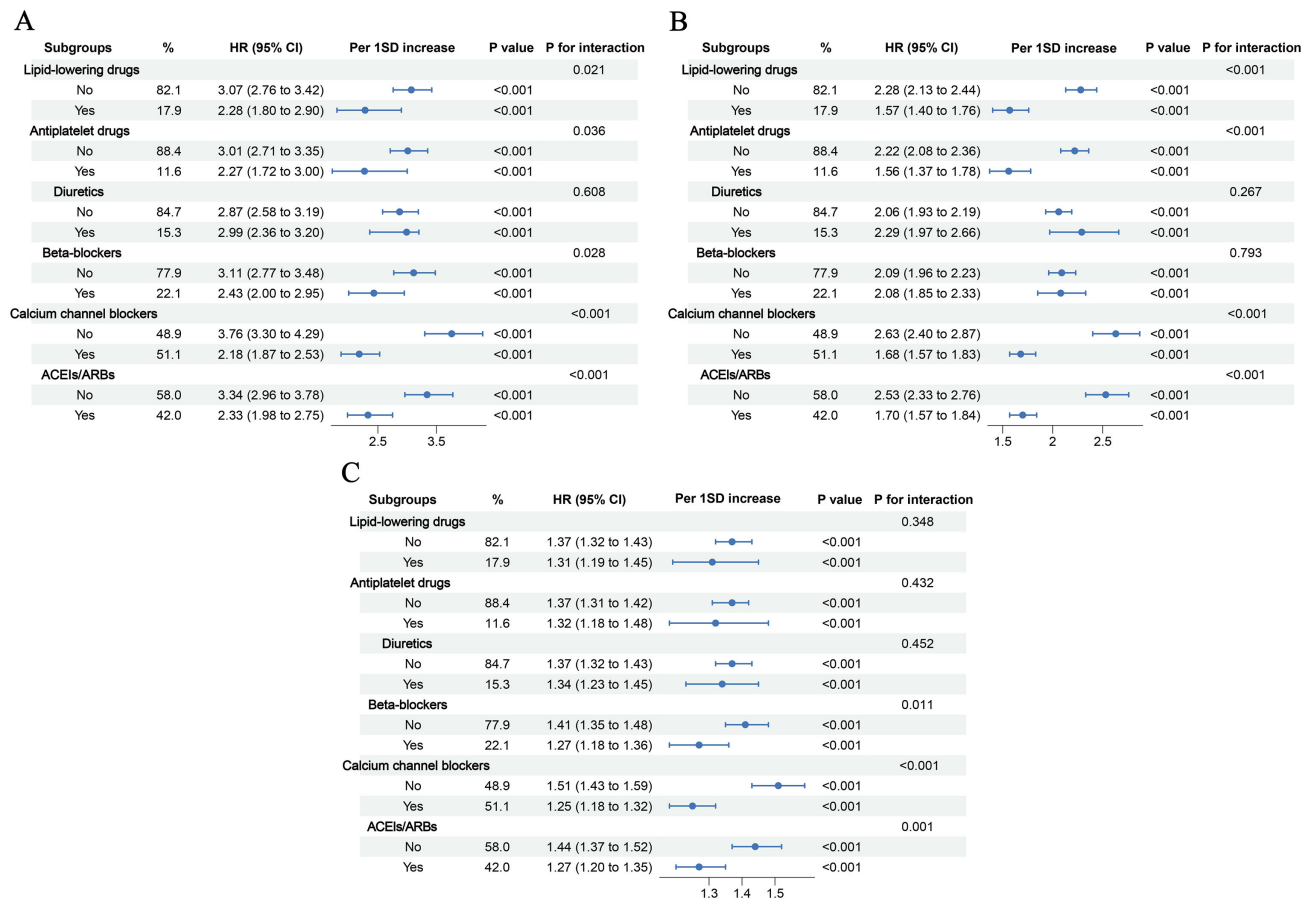
Subgroup analyses based on medication use **(A)** PLR; **(B)** PIV; **(C)** IBI.

Recognizing that individual patient differences may influence the progression from hypertension to future diabetes, we performed a series of subgroup analyses to assess the robustness of our findings. First, to account for the potential impact of participants’ physical condition and disease status, we conducted subgroup analyses based on these factors. The results consistently showed that elevated levels of various composite inflammatory indices were associated with an increased risk of future diabetes in hypertensive patients across all subgroups (**Figure 7**). Furthermore, given that individuals with hypertension are often on corresponding medications, we stratified the analysis by different medication uses and re-evaluated the data. The results remained unchanged (**Figure 8**). These subgroup analyses further reinforce the credibility of our conclusions, demonstrating that the observed association is independent of any subgroup-defining factors.

## 4 Discussion

Hypertension, as a highly prevalent chronic condition, involves not only metabolic disturbances but also a persistent, low-grade systemic inflammatory state. This chronic inflammation is a critical factor in elevating blood pressure and causing vascular damage that leads to cardiovascular and cerebrovascular diseases. Furthermore, it can induce insulin resistance, thereby significantly increasing the risk of incident diabetes. Consequently, greater clinical attention should be paid to the inflammatory burden in patients with hypertension.

To address this, our study employed a multicenter cohort design to investigate the impact of several novel composite inflammatory indices—which may better reflect the overall inflammatory load than traditional single markers—on the future risk of diabetes in hypertensive individuals. Our findings reveal that elevated levels of all assessed composite inflammatory indices were significantly associated with an increased risk of new-onset diabetes, exhibiting clear dose-response relationships and specific effect thresholds. Notably, this escalating risk became particularly pronounced when the PLR, PIV, and IBI exceeded 122.75, 142.01, and 5.19, respectively. Among these three indices, the IBI demonstrated the most prominent predictive capability, suggesting its potential as a simple and reliable clinical risk assessment tool. Furthermore, the threshold analysis implies that controlling inflammation to a lower level in hypertensive patients at an early stage may not only help reduce the incidence of cardiovascular and cerebrovascular events but also contribute to lowering the long-term risk of diabetes. In summary, this study provides a crucial theoretical foundation for emphasizing and managing inflammation in hypertensive patients within clinical practice, while also offering a novel perspective for the early prevention of diabetes.

Currently, these composite inflammatory indices have been widely applied in the diagnosis, prediction, and prognosis assessment of various diseases, demonstrating considerable efficacy (31, 41–44). This body of evidence indirectly but robustly supports the concept of a sustained chronic inflammatory state in patients with hypertension (36, 41, 42, 44). For instance, within the hypertensive population, certain composite inflammatory indices have been closely linked to the development of non-alcoholic fatty liver disease and bone loss, thereby corroborating the persistent low-grade inflammatory milieu present in these individuals (41, 42). In the field of oncology, the prognostic value of these indices is particularly notable. Inflammatory burden markers, exemplified by the IBI, are considered to more comprehensively capture the overall inflammatory response elicited by the tumor and its microenvironment (33, 45). Elevated IBI levels are often intimately associated with tumor progression, and research suggests it serves as an effective prognostic indicator for gastrointestinal cancers (31, 33, 45, 46). Similarly, the PIV, which integrates inflammatory and immune status, demonstrates considerable predictive power (30, 32, 47, 48). Studies have confirmed that PIV is not only associated with metabolic conditions like vascular calcification and dyslipidemia but its elevation may also signal an increased risk of mortality in patients with infections (30, 32, 48). In summary, the aforementioned studies collectively reinforce the reality of a chronic inflammatory state in hypertension and highlight the significant potential of these convenient, easily accessible composite inflammatory indices in clinical evaluation. Their broad applicability provides a solid foundation for our investigation into the value of these indices for predicting diabetes risk in the hypertensive population.

Although our study demonstrates a significant association between elevated composite inflammatory indices, particularly IBI, and an increased risk of incident diabetes in patients with hypertension, the underlying mechanisms remain to be fully elucidated. First, chronic inflammation induces insulin resistance, the central link between hypertension and diabetes (49, 50). Inflammatory factors (such as TNF-α and IL-6) interfere with insulin receptor substrate phosphorylation, inhibiting insulin signaling and reducing glucose uptake in peripheral tissues (50–52). Second, vascular endothelial dysfunction plays a key role. Hypertension damages the endothelium, and inflammation exacerbates this damage (53, 54). Reduced nitric oxide bioavailability impairs vasodilation and diminishes insulin delivery to target tissues, worsening glucose metabolism (55–57). Third, oxidative stress and inflammation form a vicious cycle (58, 59). Elevated reactive oxygen species directly damage pancreatic β-cells, impairing insulin secretion, while also activating inflammatory pathways that produce more inflammatory factors (58–60). Finally, inflammation alters adipokine profiles, causing adipose tissue dysfunction that promotes pro-inflammatory adipokines (e.g., leptin) and reduces insulin-sensitizing adiponectin, further disrupting metabolic balance (61–63). In summary, these interconnected mechanisms collectively form the pathological bridge linking chronic inflammation to diabetes in hypertensive patients.

This study has several strengths. First, it reveals for the first time a significant association between composite inflammatory indices and the risk of diabetes in patients with hypertension. This finding expands the research perspective on diabetes prevention and management in the hypertensive population, potentially offering important clinical implications for risk screening and early intervention in high-risk individuals. Second, the study is based on a large sample size and employs systematic statistical analyses, which enhances the reliability and accuracy of the findings to a certain extent.

Nevertheless, this study also has inherent limitations. First, although this was a multicenter study with a substantial sample size, all participants were of Chinese descent. Therefore, whether the findings can be generalized to other countries or ethnic populations remains to be validated. Second, this study only considered baseline data for the inflammatory indices and did not capture their dynamic changes during follow-up, thus precluding assessment of how evolving inflammatory status affects diabetes risk. Third, due to data source limitations, we lacked detailed information on participants’ dietary habits, physical activity levels, and other lifestyle factors that may potentially confound the development of diabetes. Future studies should incorporate these variables for comprehensive analysis. Fourth, although this study employed a longitudinal cohort design, it cannot definitively establish a causal relationship between composite inflammatory indices and diabetes risk. Further basic research and interventional trials are needed to confirm these findings.

## 5 Conclusion

This study found that inflammatory levels in patients with hypertension are significantly associated with an increased risk of diabetes, and that various composite inflammatory indices exhibit good predictive ability. Among these, the IBI demonstrated the strongest predictive performance. This finding expands the research perspective on diabetes prevention and management in the hypertensive population. Given that these composite inflammatory indices are easily accessible and cost-effective, they hold promise for integration into routine clinical assessment of hypertensive patients in the future. Such application could provide valuable tools for diabetes risk screening, early prevention, and individualized management, thereby offering potential clinical utility.

## Data Availability

The raw data supporting the conclusions of this article will be made available by the authors, without undue reservation.
