# Supplementary material for: Multicenter cohort study reveals: composite inflammatory indexs are associated with increased risk of diabetes in patients with hypertension
Source: Front Endocrinol (Lausanne). 2026 Apr 21;17:1819071. doi: 10.3389/fendo.2026.1819071 (PMC13138947; doi:10.3389/fendo.2026.1819071)
Supplement: Supplementary file 1 [file SupplementaryFile1.docx]

**Table S1**.Using data from Suining Central Hospital as external data to validate the threshold effect of composite inflammatory markers on diabetes risk in hypertensive patients

| **Diabetes** | Model 1 | Model 2 | Model 3 | Model 4 |
| --- | --- | --- | --- | --- |
|  | HR (95% CI) P | HR (95% CI) P | HR (95% CI) P | HR (95% CI) P |
| **PLR** |  |  |  |  |
| Turning point (ng/dL) | 122.75 | 122.75 | 122.75 | 122.75 |
| <= 122.75 | Reference | Reference | Reference | Reference |
| > 122.75 | 2.302 [2.019, 2.624]  <0.001 | 2.273 [1.992, 2.593]  <0.001 | 2.170 [1.899, 2.478]  <0.001 | 2.159 [1.891, 2.466]  <0.001 |
| **PIV** |  |  |  |  |
| Turning point (ng/dL) | 142.01 | 142.01 | 142.01 | 142.01 |
| <= 142.01 | Reference | Reference | Reference | Reference |
| > 142.01 | 2.305 [2.021, 2.625]  <0.001 | 2.275 [1.993, 2.594]  <0.001 | 2.172 [1.901,2.4789]  <0.001 | 2.163 [1.893, 2.469]  <0.001 |
| **IBI** |  |  |  |  |
| Turning point (ng/dL) | 5.19 | 5.19 | 5.19 | 5.19 |
| <= 5.19 | Reference | Reference | Reference | Reference |
| > 5.19 | 2.307 [2.023, 2.628]  <0.001 | 2.303 [2.019, 2.625]  <0.001 | 2.275 [1.993, 2.594]  <0.001 | 2.174 [1.902, 2.481]  <0.001 |

Model 1: no covariates were adjusted.

Model 2: age, sex, BMI, smoking status, drinking status, SBP, and DBP were adjusted.

Model 3: Model 2 plus adjustment for TSH, ALT, AST, TC, TG, HDL.C, LDL.C, and FPG.

Model 4: Model 3 plus adjustment for CHD, Hyperlipidemia, Lipid-lowering drugs, Antiplatelet drug, diuretics, beta-blockers, ACEIs/ARBs, and calcium channel blockers.

Abbreviations: PLR, platelet-to-lymphocyte ratio; PIV, pan-immune-inflammation valu; IBI, inflammatory burden index; HR, hazard ratio; CI, confidence interval

Other abbreviations, see Table 1.

**Table S2**. Using age stratification to validate the threshold effect of composite inflammatory markers on diabetes risk in hypertensive patients

| **Diabetes** | Model 1 | Model 2 | Model 3 | Model 4 |
| --- | --- | --- | --- | --- |
|  | HR (95% CI) P | HR (95% CI) P | HR (95% CI) P | HR (95% CI) P |
| **PLR** |  |  |  |  |
| Age<65 |  |  |  |  |
| Turning point (ng/dL) | 122.75 | 122.75 | 122.75 | 122.75 |
| <= 122.75 | Reference | Reference | Reference | Reference |
| > 122.75 | 2.227 [2.013, 2.464]  <0.001 | 2.182 [1.969, 2.418]  <0.001 | 2.172 [1.962, 2.404]  <0.001 | 2.163 [1.959, 2.388]  <0.001 |
| Age>=65 |  |  |  |  |
| Turning point (ng/dL) | 122.75 | 122.75 | 122.75 | 122.75 |
| <= 122.75 | Reference | Reference | Reference | Reference |
| > 122.75 | 2.441 [2.049, 2.907]  <0.001 | 2.269 [1.900, 2.710]  <0.001 | 2.260 [1.892, 2.700]  <0.001 | 2.236 [1.865, 2.681]  <0.001 |
| **PIV** |  |  |  |  |
| Age<65 |  |  |  |  |
| Turning point (ng/dL) | 142.01 | 142.01 | 142.01 | 142.01 |
| <= 142.01 | Reference | Reference | Reference | Reference |
| > 142.01 | 2.232 [2.017, 2.469]  <0.001 | 2.186 [1.972, 2.422]  <0.001 | 2.176 [1.966, 2.409]  <0.001 | 2.168 [1.963, 2.394]  <0.001 |
| **PIV** |  |  |  |  |
| Age>=65 |  |  |  |  |
| Turning point (ng/dL) | 142.01 | 142.01 | 142.01 | 142.01 |
| <= 142.01 | Reference | Reference | Reference | Reference |
| > 142.01 | 2.441 [2.049, 2.907]  <0.001 | 2.269 [1.900, 2.710]  <0.001 | 2.260 [1.892, 2.700]  <0.001 | 2.239 [1.868, 2.685]  <0.001 |
| **IBI** |  |  |  |  |
| Age<65 |  |  |  |  |
| Turning point (ng/dL) | 5.19 | 5.19 | 5.19 | 5.19 |
| <= 5.19 | Reference | Reference | Reference | Reference |
| > 5.19 | 2.159 [1.948, 2.392]  <0.001 | 2.158 [1.948, 2.391]  <0.001 | 2.149 [1.942, 2.378]  <0.001 | 2.139 [1.937, 2.362]  <0.001 |
| Age>=65 |  |  |  |  |
| Turning point (ng/dL) | 5.19 | 5.19 | 5.19 | 5.19 |
| <= 5.19 | Reference | Reference | Reference | Reference |
| > 5.19 | 2.422 [2.034, 2.884]  <0.001 | 2.252 [1.886, 2.689]  <0.001 | 2.243 [1.878, 2.679]  <0.001 | 2.222 [1.853, 2.663]  <0.001 |

Model 1: no covariates were adjusted.

Model 2: age, sex, BMI, smoking status, drinking status, SBP, and DBP were adjusted.

Model 3: Model 2 plus adjustment for TSH, ALT, AST, TC, TG, HDL.C, LDL.C, and FPG.

Model 4: Model 3 plus adjustment for CHD, Hyperlipidemia, Lipid-lowering drugs, Antiplatelet drug, diuretics, beta-blockers, ACEIs/ARBs, and calcium channel blockers.

Abbreviations: PLR, platelet-to-lymphocyte ratio; PIV, pan-immune-inflammation valu; IBI, inflammatory burden index; HR, hazard ratio; CI, confidence interval

Other abbreviations, see Table 1.

**Table S3**. Using BMI stratification to validate the threshold effect of composite inflammatory markers on diabetes risk in hypertensive patients

| **Diabetes** | Model 1 | Model 2 | Model 3 | Model 4 |
| --- | --- | --- | --- | --- |
|  | HR (95% CI) P | HR (95% CI) P | HR (95% CI) P | HR (95% CI) P |
| **PLR** |  |  |  |  |
| BMI<24 |  |  |  |  |
| Turning point (ng/dL) | 122.75 | 122.75 | 122.75 | 122.75 |
| <= 122.75 | Reference | Reference | Reference | Reference |
| > 122.75 | 2.413 [2.040, 2.853]  <0.001 | 2.396 [2.020, 2.841]  <0.001 | 2.333 [1.963, 2.773]  <0.001 | 2.320 [1.947, 2.763]  <0.001 |
| BMI>=24 |  |  |  |  |
| Turning point (ng/dL) | 122.75 | 122.75 | 122.75 | 122.75 |
| <= 122.75 | Reference | Reference | Reference | Reference |
| > 122.75 | 2.188 [1.975, 2.424]  <0.001 | 2.169 [1.955, 2.407]  <0.001 | 2.151 [1.941, 2.383]  <0.001 | 2.138 [1.933, 2.364]  <0.001 |
| **PIV** |  |  |  |  |
| BMI<24 |  |  |  |  |
| Turning point (ng/dL) | 142.01 | 142.01 | 142.01 | 142.01 |
| <= 142.01 | Reference | Reference | Reference | Reference |
| > 142.01 | 2.413 [2.040, 2.853]  <0.001 | 2.396 [2.020, 2.841]  <0.001 | 2.333 [1.963, 2.773]  <0.001 | 2.320 [1.947, 2.763]  <0.001 |
| **PIV** |  |  |  |  |
| BMI>=24 |  |  |  |  |
| Turning point (ng/dL) | 142.01 | 142.01 | 142.01 | 142.01 |
| <= 142.01 | Reference | Reference | Reference | Reference |
| > 142.01 | 2.193 [1.979, 2.429]  <0.001 | 2.174 [1.959, 2.412]  <0.001 | 2.155 [1.945, 2.388]  <0.001 | 2.143 [1.937, 2.370]  <0.001 |
| **IBI** |  |  |  |  |
| BMI<24 |  |  |  |  |
| Turning point (ng/dL) | 5.19 | 5.19 | 5.19 | 5.19 |
| <= 5.19 | Reference | Reference | Reference | Reference |
| > 5.19 | 2.413 [2.040, 2.853]  <0.001 | 2.396 [2.020, 2.841]  <0.001 | 2.333 [1.963, 2.773]  <0.001 | 2.320 [1.947, 2.763]  <0.001 |
| BMI>=24 |  |  |  |  |
| Turning point (ng/dL) | 5.19 | 5.19 | 5.19 | 5.19 |
| <= 5.19 | Reference | Reference | Reference | Reference |
| > 5.19 | 2.158 [1.948, 2.390]  <0.001 | 2.140 [1.929, 2.375]  <0.001 | 2.122 [1.916, 2.351]  <0.001 | 2.108 [1.907, 2.331]  <0.001 |

Model 1: no covariates were adjusted.

Model 2: age, sex, BMI, smoking status, drinking status, SBP, and DBP were adjusted.

Model 3: Model 2 plus adjustment for TSH, ALT, AST, TC, TG, HDL.C, LDL.C, and FPG.

Model 4: Model 3 plus adjustment for CHD, Hyperlipidemia, Lipid-lowering drugs, Antiplatelet drug, diuretics, beta-blockers, ACEIs/ARBs, and calcium channel blockers.

Abbreviations: PLR, platelet-to-lymphocyte ratio; PIV, pan-immune-inflammation valu; IBI, inflammatory burden index; HR, hazard ratio; CI, confidence interval

Other abbreviations, see Table 1.
